# The prevalence of osteoporosis in China, a community based cohort study of osteoporosis

**DOI:** 10.3389/fpubh.2023.1084005

**Published:** 2023-02-16

**Authors:** Jing Wang, Bing Shu, De-zhi Tang, Chen-guang Li, Xing-wen Xie, Li-juan Jiang, Xiao-bing Jiang, Bo-lai Chen, Xin-chao Lin, Xu Wei, Xiang-yang Leng, Zhang-yu Liao, Bao-lin Li, Yan Zhang, Xue-jun Cui, Qing Zhang, Sheng Lu, Qi Shi, Yong-jun Wang

**Affiliations:** ^1^Longhua Hospital, Shanghai University of Traditional Chinese Medicine, Shanghai, China; ^2^Key Laboratory of Theory and Therapy of Muscles and Bones, Ministry of Education, Shanghai, China; ^3^Shanghai Geriatric Institute of Chinese Medicine, Shanghai, China; ^4^Shanghai University of Traditional Chinese Medicine, Shanghai, China; ^5^Spine Institute, Shanghai Academy of Traditional Chinese Medicine, Shanghai, China; ^6^Gansu Provincial Hospital of Traditional Chinese Medicine, Lanzhou, China; ^7^Yunnan Provincial Hospital of Traditional Chinese Medicine, Kunming, China; ^8^The First Hospital Affiliated to Guangzhou University of Traditional Chinese Medicine, Guangzhou, China; ^9^Guangdong Provincial Hospital of Traditional Chinese Medicine, Guangzhou, China; ^10^Dongzhimen Hospital, Beijing University of Chinese Medicine, Beijing, China; ^11^Wangjing Hospital, China Academy of Chinese Medical Sciences, Beijing, China; ^12^Hospital Affiliated to Changchun University of Traditional Chinese Medicine, Changchun, China; ^13^Ganzhou Nankang District Traditional Chinese Medicine Hospital, Ganzhou, China; ^14^Shenzhen Pingle Orthopaedic Hospital, Shenzhen, China; ^15^The First People's Hospital of Nankang District, Ganzhou, China

**Keywords:** prevalence, osteoporosis, China, epidemiology, risk factors, cross-sectional study

## Abstract

**Background:**

Osteoporosis has already been a growing health concern worldwide. The influence of living area, lifestyle, socioeconomic, and medical conditions on the occurrence of osteoporosis in the middle-aged and elderly people in China has not been fully addressed.

**Methods:**

The study was a multicenter cross-sectional study on the middle-aged and elderly permanent residents, which gathered information of 22,081 residents from June 2015 to August 2021 in seven representative regions of China. The bone mineral density of lumbar vertebrae and hip were determined using the dual-energy X-ray absorptiometry densitometer instruments. Serum levels of bone metabolism markers were also measured. Information about education, smoking, and chronic diseases were also collected through face-to-face interviews. Age-standardized prevalence and 95% confidence intervals (CIs) of osteopenia and osteoporosis by various criteria were estimated by subgroups and overall based on the data of China 2010 census. The relationships between the osteoporosis or osteopenia and sociodemographic variables or other factors were examined using univariate linear models and multivariable multinomial logit analyses.

**Results:**

After screening, 19,848 participants (90%) were enrolled for the final analysis. The age-standardized prevalence of osteoporosis was estimated to be 33.49%(95%CI, 32.80–34.18%) in the middle-aged and elderly Chinese permanent residents, for men and women was 20.73% (95% CI, 19.58–21.87%) and 38.05% (95% CI, 37.22–38.89%), respectively. The serum concentrations of bone metabolic markers, and calcium and phosphorus metabolism were influenced by age, body mass index (BMI), gender, education level, regions, and bone mass status. Women, aged 60 or above, BMI lower than 18.5 kg/m^2^, low education level including middle school, primary school and no formal education as well as current regular smoking, a history of fracture were all significantly associated with a higher risk of osteoporosis and osteopenia in the middle-aged and elderly people.

**Conclusions:**

This study revealed dramatic regional differences in osteoporosis prevalence in China, and female, aged 60 or older, low BMI, low education level, current regular smoking, and a history of fracture were associated with a high risk of osteoporosis. More prevention and treatment resources should be invested into particular population exposed to these risk factors.

## Introduction

Osteoporosis is a systemic skeletal disease characterized by loss of bone mass and micro architectural integrity that lead to increased bone fragility and risk of fracture (1). The prevalence of osteoporosis has increased over the past years in China (2). According to the recent multicenter survey, the age-standardized prevalence of osteoporosis in China was 6.46 and 29.13% for men and women aged 50 years and older, respectively, and the bone mineral density (BMD) values differed by demographic characteristics (3). Furthermore, ~2.33 million osteoporotic fractures occurred in 2010 in China, which was estimated to double by 2035 (4).

China is one of the most populous countries in the world with vast territory, and the prevalence and risk factors of osteoporosis varies in different regions of China. With the development of science and technology, people's life quality and health status have been significantly improved in the past decades. However, there are still huge differences in living style, socioeconomic conditions and medical status in different regions of China, such as smoking, education level, economic disparity, and chronic diseases (5–12), which may contribute to the different prevalence of osteoporosis.

To address this issue, we undertook the China Community-based Cohort of Osteoporosis (CCCO) to assess the prevalence of osteoporosis and osteopenia as well as associated risk factors.

## Methods and analysis

### Recruitment of the participants

The study, which was a multicenter cross-sectional study, was a part of the registered protocol at Clinical trials.gov (NCT02958020). The protocol was approved by the Institutional Review Board at Longhua Hospital affiliated to the Shanghai University of Traditional Chinese Medicine (2016LCSY065), and was performed in accordance with the ethical standards laid down in the 1964 Declaration of Helsinki and its later amendments or comparable ethical standards. All participants provided written informed consent before participation.

**Figure 1:**
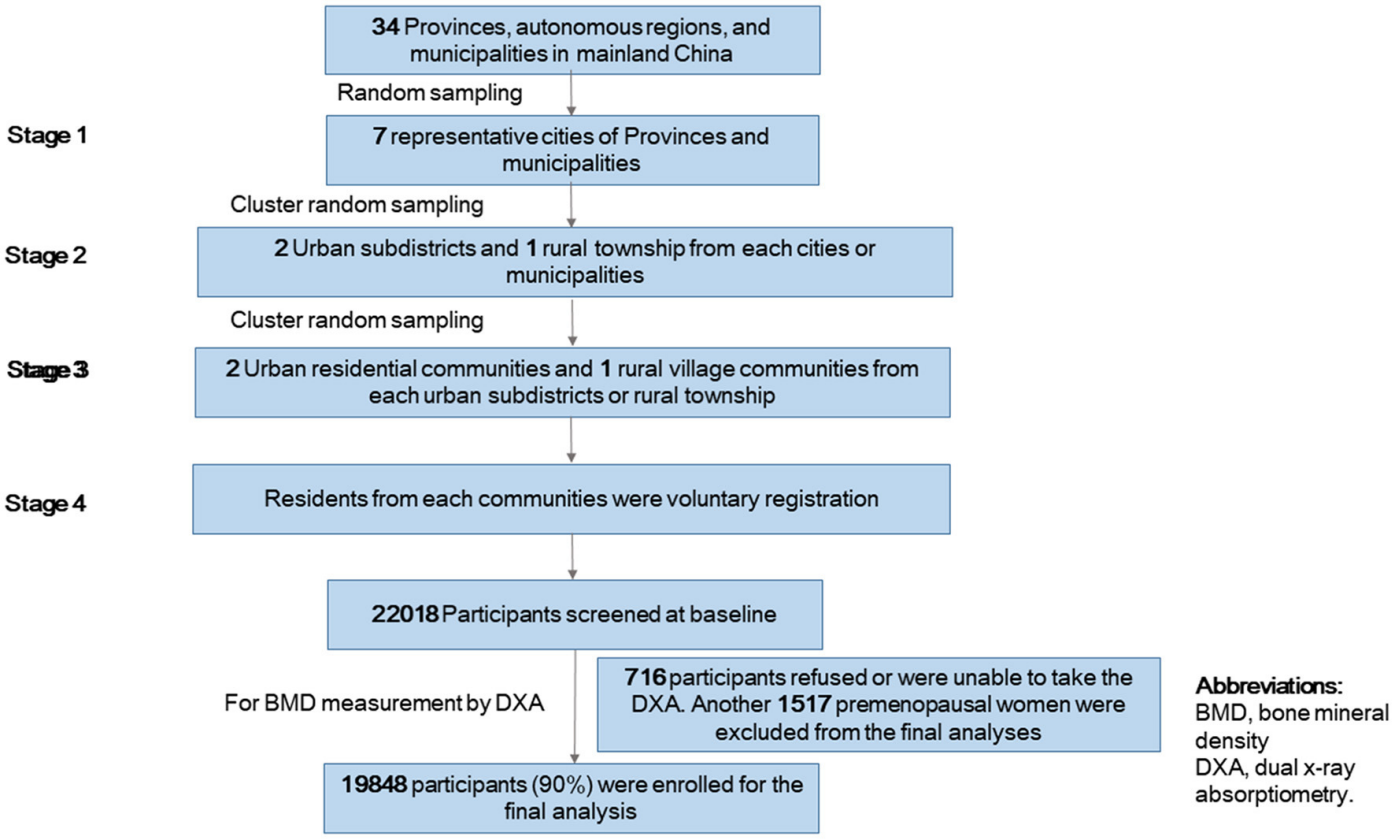
Flowchart of participant selection.

We used a multistage stratified cluster random sampling method to enroll a sample of people who would be representative of adults in China (Figure 1). In stage 1, we randomly selected seven provinces in China, including Shanghai (east), Guangdong (south), Gansu (west), Beijing (north), Jilin (north-east), Yunnan (south-west) and Jiangxi (south-west). In stage 2, we used a cluster random sampling method to select 2 urban subdistricts and 1 rural township from each cities or municipalities. In stage 3, we used cluster random sampling method to select 2 urban residential communities and 1 rural village communities from each urban subdistricts or rural township. In stage 4, residents from each community were voluntary registration. Eligible participants were permanent residents including women aged ≥40 years old and men aged ≥50 years old. Lactating or pregnant women and residents with mental health problems, acute infectious diseases and severe physical diseases who could not cooperate with the investigations were excluded. Totally 22,081 residents responded and signed the informed consent forms from June 2015 to August 2021.

### Questionnaires and physical examinations

All the participants completed paper-based questionnaires through face-to-face interviews. The questionnaires contained information about age, gender, province, education level, smoking status, history of fracture and chronic diseases including hyperlipidemia, hypertension, and diabetes. Education level was graded into six categories: no formal education, primary school, middle school, high school, college, university, or higher education. Smoking status was graded into four categories: never smoke, former smoker, current regular smoker, or passive smoker. Information about bone fracture history and chronic diseases was obtained from the outpatient and emergency treatment record books provided by the participants.

### Physical examinations

Barefoot body weight with indoor clothing and height were measured. Body mass index (BMI) was calculated as weight (kg)/height squared (m^2^). BMI was categorized into: underweight, BMI <18.5 kg/m^2^; normal weight, 18.5–24.9 kg/m^2^; overweight, ≥25 kg/m^2^.

### Measurement of serum bone metabolism markers and calcium and phosphorus metabolism indicators

After an overnight fasting, the venous blood samples of all the participants were collected in non-EDTA tubes. Within 2 h after the blood collection, the blood samples were centrifuged at 3,000 rpm for 15 min at room temperature to separate the serum. Serum concentrations of N-terminal propeptide of type I collagen (PINP), β-C-terminal telopeptide of type I collagen (β-CTX), osteocalcin (OST) were measured through an electrochemiluminescence immunoassay. Serum alkaline phosphatase (ALP) concentration was measured through a continuous monitoring technique. Total 25(OH)D [25(OH)D_3_ and 25(OH)D_2_] was detected using a sensitive and specific high-performance liquid chromatography–tandem mass spectrometry (HPLC–MS/MS) method, and parathormone (PTH) was detected through a chemiluminescence immunoassay. The o-cresolphtalein-complexone method and phosphomolybdate ultraviolet colorimetry were applied to detect total calcium (Ca) and phosphorus (P), respectively.

### Assessment of BMD and osteoporosis diagnosis

The BMD values of each single lumbar vertebra (L1–L4), the total lumbar vertebra (L1–4) and the total left hip joint (femoral neck, trochanter, and intertrochanteric region) were measured using the dual-energy X-ray absorptiometry densitometer (DXA, Hologic Discovery CI, Bedford, MA, USA) instruments. The instruments used in all the centers were of the same model, and passed the annual verification. Daily calibration program was performed each time the instrument was powered on.

For osteoporosis diagnosis, the BMD values were expressed as *T*-scores (number of standard deviations above/below the mean peak BMD of healthy young-adults of the same race and same gender). According to the Guideline for diagnosis and treatment of primary osteoporosis issued by the Chinese Society of Osteoporosis and Bone Mineral Research in 2017, participants with *T*-scores of any site ≥-1.0 were considered not having osteoporosis or osteopenia. Participants with *T*-scores of more than −2.5, and <-1.0 were diagnosed as having osteopenia, and those with *T*-score ≤−2.5 as having osteoporosis (13).

### Statistical analysis

As first step, the characteristics of the participants were shown as the mean and standard deviation, median and the interquartile range (IQR) or number and proportion in the overall population and in subgroups of gender. The bone metabolic indicators were shown as the mean and standard deviation.

Age-standardized prevalence and 95% confidence intervals (CIs) of osteopenia and osteoporosis by various criteria were estimated by subgroups and overall based on the data of China 2010 census.

The relationships between the osteoporosis or osteopenia and sociodemographic variables or other factors were examined using univariate linear models separately. A multivariable multinomial logit analysis was used to examine the association of variables with the odds of osteoporosis or osteopenia. All *P*-values were 2-tailed and had not been adjusted for multiple testing. All *P*-value < 0.05 was considered statistically significant. All statistical analyses were conducted using the SAS system, version 9.3 (SAS Institute Inc), SUDAAN software, version 10.0 (Research Triangle Institute), and SPSS V.25.0 (SPSS, Chicago, Illinois, USA).

## Results

### Demographic characteristics of the participants

For further screening, 716 participants refused or were unable to take the BMD measurement. Another 1,517 premenopausal women were excluded from the final analyses because *Z*-scores, but not *T*-scores of BMD are used for the diagnosis of osteoporosis in premenopausal women. Finally, 19,848 participants (90%) were enrolled for the final analysis. The general characteristics of the study population were presented in Table 1. The average age of all the participants was 63.79 ± 7.11, among which 28.43% were men. BMD values of men in both lumbar spine and hip were higher than those of women (*P* < 0.001). The proportion of osteoporosis diagnosed by hip BMD was significantly lower than that by lumbar spine BMD (*P* < 0.001).

**Table 1:** General characteristics of the participants.

	**Total**	**Male**	**Female**	***P*-value**
	**(*****n*** = **19,848)**	**(*****n*** = **5,643)**	**(*****n*** = **14,205)**	
Age (year), mean (SD)	63.79 (7.11)	65.40 (6.97)	63.16 (7.06)	<0.001
**Age, n (%)**				
40–49 years	276 (1.39)	67 (1.19)	209 (1.47)	<0.001
50–59 years	5,446 (27.44)	1,031 (18.27)	4,415 (31.08)	
60–69 years	9,740 (49.07)	2,925 (51.83)	6,815 (47.98)	
70–79 years	4,244 (21.38)	1,561 (27.66)	2,683 (18.89)	
80- years	142 (0.72)	59 (1.05)	83 (0.58)	
BMI^a^ (kg/m^2^), median (P25, P75; n = 19,459)	24.02 (21.99, 26.17)	24.38 (22.49, 26.40)	23.83 (21.79, 26.06)	<0.001
**BMI**^a^**, n (%) (*****n*** = **19,459)**				
<18.5 kg/m^2^	598 (3.07)	128 (2.32)	470 (3.37)	<0.001
18.5–24.9 kg/m^2^	11,570 (59.46)	3,064 (55.59)	8,506 (60.99)	
≥25.0 kg/m^2^	7,291 (37.47)	2,320 (42.09)	4,971 (35.64)	
**Education**^b^ **(n** = **18,877)**				
No formal education	1,035 (5.48)	98 (1.85)	937 (6.90)	<0.001
Primary school	2,915 (15.44)	760 (14.34)	2,155 (15.87)	
Middle school	5,706 (30.23)	1,676 (31.62)	4,030 (29.68)	
High school	6,104 (32.34)	1,459 (27.52)	4,645 (34.21)	
College	1,927 (10.21)	707 (13.34)	1,220 (8.99)	
University or higher	1,190 (6.30)	601 (11.34)	589 (4.34)	
**Smoking status**^c^ **(n** = **18,696)**				
Never	14,444 (77.26)	2,589 (47.97)	11,855 (89.15)	<0.001
Former	1,007 (5.39)	910 (16.84)	97 (0.73)	
Current regular	1,996 (10.68)	1,732 (32.07)	265 (1.99)	
Passive smoking	1,249 (6.68)	168 (3.11)	1,081 (8.13)	
**Region, n (%)**				
Shanghai	5,810 (29.27)	1,740 (30.84)	4,069 (28.64)	<0.001
Beijing	2,784 (14.03)	762 (13.51)	2,022 (14.23)	
Guangdong	3,376 (17.01)	813 (14.41)	2,563 (18.04)	
Jilin	1,626 (8.19)	443 (7.85)	1,183 (8.33)	
Gansu	2,724 (13.72)	707 (12.53)	2,017 (14.20)	
Yunnan	1,780 (8.97)	692 (12.27)	1,088 (7.66)	
Jiangxi	1,748 (8.81)	485 (8.60)	1,263 (8.89)	
History of fracture^d^, *n* (%)	3,657 (18.44)	944 (16.75)	2,713 (19.11)	<0.001
Hyperlipidemia, *n* (%)	3,810 (19.20)	966 (17.12)	2,844 (20.02)	<0.001
Hypertension, *n* (%)	6,729 (33.90)	2,036 (36.07)	4,693 (33.04)	<0.001
Diabetes, *n* (%)	2,352 (11.85)	791 (14.00)	1,561 (10.99)	<0.001
Lumbar BMD^e^, mean (SD)	0.86 (0.17)	0.96 (0.17)	0.83 (0.15)	<0.001
Hip BMD^f^, mean (SD)	0.81 (0.14)	0.89 (0.14)	0.77 (0.13)	<0.001
**Bone mass category** ^g^ **, n (%)**				
Osteoporosis	6,599 (33.39)	1,090 (19.39)	5,509 (38.96)	<0.001
Osteopenia	7,535 (38.13)	2,042 (36.31)	5,493 (38.85)	
**Bone mass category** ^h^ **, n (%)**				
Osteoporosis	2,032 (11.85)	213 (4.36)	1,819 (14.85)	<0.001
Osteopenia	8,126 (47.41)	2,083 (42.60)	6,043 (49.33)	
**Bone mass category** ^i^ **, n (%)**				
Osteoporosis	7,051 (35.52)	1,151 (20.40)	5,900 (41.53)	<0.001
Osteopenia	8,330 (41.97)	2,464 (43.65)	5,866 (41.30)	

^a^There were 389 missing values for body mass index (BMI).

^b^There were 971 missing values for education level.

^c^There were 1,152 missing values for smoking status.

^d^There were 13 missing values for history of fracture.

^e^There were 197 missing values for lumbar BMD.

^f^There were 197 missing values for hip BMD.

^g^Diagnosed by lumbar spine BMD.

^h^Diagnosed by hip BMD.

^i^Diagnosed by BMD of lumbar spine or hip.

### Serum levels of bone metabolic markers and calcium and phosphorus metabolism indicators

The serum concentrations of bone metabolic markers, and calcium and phosphorus metabolism indicators listed in the Supplementary Table S1 were influenced by age, BMI, gender, education level, regions and bone mass status. Among women, the serum concentrations of P, Ca, PINP, β-CTX, OST, ALP, and PTH were significantly higher than those of men, while 25(OH)D of men was higher than that of women (*P* < 0.001). The expression levels of these markers and indicators also showed significant differences among people of different ages (*P* < 0.001). Serum levels of PINP and OST as well as Ca, P, and 25(OH)D showed peak values in the participants of 50–59 years old, followed by gradual decreasing trends with aging, while bone resorption marker β-CTX showed a continuous increasing trend along with intensified aging process. Serum levels of PTH and another bone formation marker ALP also displayed upward trends until the years of 70–79. As BMI increased, the serum concentrations of P, PINP, OST, β-CTX, and 25(OH)D decreased (*P* < 0.001), while ALP and PTH increased (*P* < 0.001) suggesting relative higher bone turnover of people with lower BMI. Along with increased education level, serum concentrations of PINP, OST, and ALP showed overall downward trends indicating decreased bone formation in people with higher education level. The serum levels of these markers and indicators were also different among people of different regions (*P* < 0.001). Shanghai had the lowest concentration of serum PINP and the highest concentration of serum β-CTX, and Jiangxi had the highest concentration of serum PINP and OST. Guangdong showed the lowest concentration of serum ALP and PTH and the highest 25(OH)D, and Gansu had the highest ALP and lowest 25(OH)D. The serum concentrations of all the bone metabolic markers as well as calcium and phosphorus in osteoporosis group were higher than those in both osteopenia group and normal group (*P* < 0.01).

### Estimated prevalence of osteoporosis in the middle aged and elderly people in China

**Figure 2:**
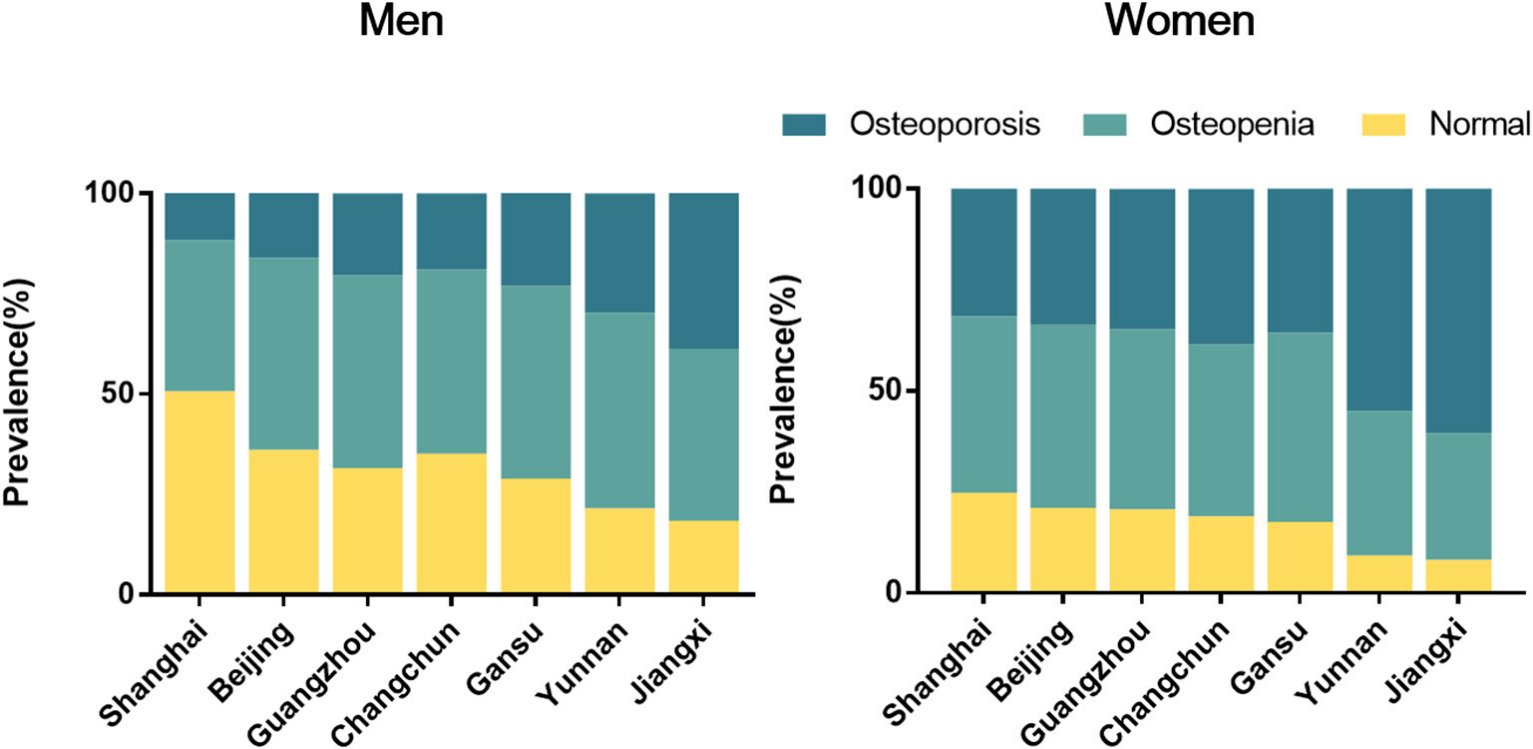
Prevalence of osteopenia and osteoporosis in the middle-aged and elderly people in different regions of China by sexual.

Estimated prevalence of osteoporosis was listed in Table 2. The age-standardized prevalence of osteoporosis was estimated to be 33.49% (95% CI, 32.80–34.18%) in the middle-aged and elderly Chinese permanent residents, 20.73% (95% CI, 19.58–21.87%) in men, and 38.05% (95% CI, 37.22–38.89%) in women. The prevalence of osteoporosis was increased with aging in women. For men, the prevalence of osteoporosis was 23.47, 19.10, 20.99, and 23.73% respectively in their 50, 60, 70, and 80 s and older. For women, the prevalence of osteoporosis was 13.39, 25.99, 38.67, 48.78, and 57.75% in their 40, 50, 60, 70, and 80 s and older (Supplementary Table S2). In addition, the prevalence of osteoporosis showed a downward trend with the increase of education level in women with education level lower than college, and the prevalence showed a similar trend with BMI in both men and women (Supplementary Table S2). There was a high prevalence of osteoporosis in current regular smoking participants in men (23.01%; 95% CI, 20.90–25.12%, Supplementary Table S2). The estimated prevalence of osteoporosis among total population also varied regionally, with the lowest prevalence in the east (Shanghai, 26.21%; 95% CI, 25.00–27.39%) and the highest in the south-east (Jiangxi, 54.58%; 95% CI, 52.06–57.10%). The prevalence of osteoporosis of the south-west (Yunnan), the west (Gansu), the north-east (Jilin), the south (Guangdong), and the north (Beijing) was 45.15, 33.49, 32.65, 31.55, and 29.11%, respectively. The respective prevalence of osteoporosis in man and women also showed the similar results (Figure 2). The prevalence of osteoporosis was also significantly increased among participants with a history of previous fractures. But a lower prevalence of osteoporosis was found in people with chronic diseases such as hypertension, hyperlipidemia, or diabetes compared with those without the chronic diseases (Supplementary Table S2).

**Table 2:** Estimated prevalence of osteoporosis and osteopenia in each group.

	**Normal**	**Osteopenia**	**Osteoporosis**
	**(*****n*** = **4,467)**	**(*****n*** = **8,330)**	**(*****n*** = **7,051)**
Overall	23.27 (22.63, 23.90)	43.24 (42.50, 43.98)	33.49 (32.80, 34.18)
**Age**			
40–49 years (*n* = 276)	35.43 (29.75, 41.11)	45.29 (39.37, 51.20)	19.29 (14.58, 23.98)
50–59 years (*n* = 5,446)	24.79 (23.64, 25.93)	46.43 (45.10, 47.75)	28.79 (27.58, 29.99)
60–69 years (*n* = 9,739)	21.91 (21.07, 22.75)	41.97 (40.97, 42.97)	36.12 (35.15, 37.09)
70–79 years (*n* = 4,244)	20.53 (19.30, 21.76)	36.88 (35.42, 38.35)	42.59 (41.08, 44.09)
80- years (*n* = 142)	18.07 (11.84, 24.30)	34.86 (26.89, 42.82)	47.07 (38.69, 55.45)
**Gender**			
Men (*n* = 5,642)	34.57 (33.25, 35.89)	44.70 (43.30, 46.11)	20.73 (19.58, 21.87)
Women (*n* = 14,205)	19.23 (18.51, 19.94)	42.72 (41.85, 43.59)	38.05 (37.22, 38.89)
**BMI**			
<18.5 kg/m^2^ (*n* = 598)	6.10 (3.90, 8.30)	26.47 (22.51, 30.43)	67.43 (63.24, 71.62)
18.5–24.9 kg/m^2^ (*n* = 11,570)	17.57 (16.81, 18.32)	43.79 (42.82, 44.77)	38.64 (37.69, 39.59)
≥25.0 kg/m^2^ (*n* = 7,291)	33.69 (32.54, 34.83)	44.11 (42.92, 45.31)	22.20 (21.23, 23.18)
**Education**			
No formal education	13.05 (10.78, 15.32)	34.60 (31.45, 37.75)	52.36 (49.07, 55.64)
Primary school	17.63 (16.13, 19.14)	40.07 (38.16, 41.98)	42.30 (40.38, 44.22)
Middle school	23.51 (22.33, 24.68)	44.99 (43.52, 46.46)	31.46 (30.21, 32.72)
High school	24.70 (23.55, 25.84)	44.76 (43.45, 46.07)	30.54 (29.34, 31.75)
College	27.61 (25.46, 29.76)	44.11 (41.74, 46,48)	28.28 (26.18, 30.39)
University or higher	27.81 (25.05, 30.57)	42.68 (39.60, 45.76)	29.51 (26.69, 32.34)
**Region**			
Shanghai	31.71 (30.42, 33.00)	42.08 (40.72, 43.45)	26.21 (25.00, 27.39)
Beijing	25.03 (23.28, 26.79)	45.86 (43.88, 47.83)	29.11 (27.35, 30.87)
Guangdong	23.10 (21.59, 24.60)	45.35 (43.59, 47.10)	31.55 (29.94, 33.17)
Jilin	23.18 (20.93, 25.43)	43.33 (40.76, 45.90)	33.49 (31.11, 35.87)
Gansu	20.33 (18.71, 21.95)	47.02 (45.03, 49.00)	32.65 (30.81, 34.49)
Yunnan	14.04 (12.25, 15.83)	40.82 (38.33, 43.31)	45.15 (42.66, 47,63)
Jiangxi	10.98 (9.34, 12.63)	34.44 (32.01, 36.86)	54.58 (52.06, 57.10)
**Smoking status**^a^ **(*****n*** = **18,695)**			
Never	24.04 (23.18, 24.9)	44.59 (43.61, 45.58)	31.37 (30.46, 32.27)
Former	40.25 (36.63, 43.86)	43.49 (39.8, 47.17)	16.26 (13.57, 18.96)
Current regular	29.88 (27.54, 32.23)	47.30 (44.74, 49.87)	22.82 (20.68, 24.96)
Passive smoking	21.81 (19.18, 24.43)	46.57 (43.41, 49.72)	31.63 (28.73, 34.53)
History of fracture (*n* = 3,657)	17.27 (15.94,18.60)	42.91 (41.19, 44.64)	39.82 (38.13, 41.50)
**Hyperlipidemia**			
Yes	26.76 (25.24,28.28)	42.57 (40.90, 44.25)	30.66 (29.13,32.20)
No	22.48 (21.79,23.18)	43.39 (42.57,44.21)	34.13 (33.35,34.90)
**Hypertension**			
Yes	27.37 (26.22,28.53)	43.19 (41.92, 44.47)	29.43 (28.28,30.57)
No	21.48 (20.72,22.24)	43.26 (42.36,44.16)	35.26 (34.40,36.11)
**Diabetes**			
Yes	31.05 (29.01,33.08)	41.30 (39.16, 43.44)	27.65 (25.74,29.57)
No	22.36 (21.70,23.03)	43.47 (42.68,44.25)	34.17 (33.43,34.91)

^a^There were 1,152 missing values for smoking status.

### Risk factors for osteoporosis and osteopenia in the middle aged and elderly people in China

In the multivariable multinomial logit models, compared with those in Shanghai, the middle-aged and elderly people in other provinces were more susceptible to osteoporosis and osteopenia. In addition, women, aged 60 or above, BMI lower than 18.5 kg/m^2^; low education level including middle school, primary school and no formal education as well as current regular smoking, a history of fracture were all significantly associated with a higher risk of osteoporosis and osteopenia in the middle-aged and elderly people. BMI equal to or lower than 25.0 kg/m^2^ and a history of diabetes were associated with lower risks of both osteoporosis and osteopenia, and a history of hypertension was associated with a lower risk of osteoporosis (Table 3).

**Table 3:** Risk factors for osteoporosis and osteopenia by univariate and multivariate logistic regression analyses.

**Risk factors**	**Osteopenia OR (95% CI)**	***P*-value^a^**	**Osteopenia OR (95% CI)**	***P*-value^b^**	**Osteoporosis OR (95% CI)**	***P*-value^a^**	**Osteoporosis OR (95% CI)**	***P*-value^b^**
**Region**								
Shanghai	1.00		1.000		1.000		1.000	
Beijing	1.398 (1.248, 1.567)	<0.001	1.607 (1.413, 1.827)	<0.001	1.476 (1.306, 1.669)	<0.001	2.088 (1.790, 2.437)	<0.001
Guangdong	1.504 (1.35, 1.677)	<0.001	1.565 (1.382, 1.772)	<0.001	1.659 (1.477, 1.863)	<0.001	1.959 (1.690, 2.270)	<0.001
Jilin	1.457 (1.261, 1.683)	<0.001	1.552 (1.326, 1.817)	<0.001	1.904 (1.639, 2.211)	<0.001	2.136 (1.779, 2.565)	<0.001
Gansu	1.727 (1.532, 1.947)	<0.001	1.917 (1.676, 2.193)	<0.001	1.950 (1.718, 2.214)	<0.001	2.702 (2.306, 3.166)	<0.001
Yunnan	2.121 (1.806, 2.49)	<0.001	2.973 (2.486, 3.557)	<0.001	3.916 (3.339, 4.594)	<0.001	6.981 (5.712, 8.533)	<0.001
Jiangxi	2.562 (2.049, 3.202)	<0.001	2.646 (2.048, 3.418)	<0.001	6.682 (5.390, 8.285)	<0.001	6.823 (5.193, 8.964)	<0.001
**Gender**								
Men	1.000		1.000		1.000		1.000	
Women	1.989 (1.841, 2.148)	<0.001	2.360 (2.123, 2.622)	<0.001	4.375 (4.000, 4.784)	<0.001	6.060 (5.289, 6.943)	<0.001
**Age, year**								
<60	1.000		1.000		1.000		1.000	
≥60	1.034 (0.955, 1.120)	0.403	1.452 (1.318, 1.599)	<0.001	1.610 (1.478, 1.755)	<0.001	2.673 (2.382, 2.998)	<0.001
**BMI, kg/m** ^2^								
<18.5	1.685 (1.141, 2.488)	0.009	1.867 (1.221, 2.857)	0.004	5.224 (3.629, 7.520)	<0.001	5.927 (3.909, 8.988)	<0.001
18.5–24.9	1.000		1.000		1.000		1.000	
≥25.0	0.544 (0.504, 0.587)	<0.001	0.506 (0.465, 0.550)	<0.001	0.306 (0.282, 0.333)	<0.0001	0.272 (0.246, 0.301)	<0.001
**Education**								
No formal education	1.867 (1.461, 2.386)	<0.001	1.728 (1.314, 2.273)	<0.001	4.151 (3.256, 5.293)	<0.001	2.765 (2.039, 3.750)	<0.001
Primary school	1.562 (1.313, 1.857)	<0.001	1.676 (1.374, 2.044)	<0.001	2.362 (1.971, 2.831)	<0.001	2.263 (1.784, 2.871)	<0.001
Middle school	1.323 (1.135, 1.542)	<0.001	1.395 (1.169, 1.664)	<0.001	1.388 (1.177, 1.636)	<0.001	1.443(1.161, 1.792)	0.001
High school	1.230 (1.056, 1.432)	0.008	1.241 (1.042, 1.478)	0.016	1.252 (1.063, 1.474)	0.007	1.217 (0.981, 1.509)	0.074
College	1.087 (0.911, 1.297)	0.353	1.089 (0.895, 1.325)	0.395	1.072 (0.886, 1.296)	0.476	1.015 (0.797, 1.293)	0.901
University or higher	1.000		1.000		1.000		1.000	
**Smoking status**								
Never	1.000		1.000		1.000		1.000	
Former	0.567 (0.488, 0.658)	<0.001	1.015 (0.855, 1.204)	0.866	0.296 (0.247, 0.355)	<0.0001	0.937 (0.774, 1.134)	0.503
Current regular	0.815 (0.726, 0.915)	0.001	1.339 (1.165, 1.540)	<0.001	0.502 (0.441, 0.572)	<0.0001	1.604 (1.341, 1.920)	<0.001
Passive smoking	1.014 (0.865, 1.188)	0.864	0.965 (0.816, 1.142)	0.678	0.939 (0.798, 1.106)	0.451	0.989 (0.786, 1.245)	0.927
**History of fracture**								
No	1.000		1.000		1.000		1.000	
Yes	1.402 (1.264, 1.555)	<0.001	1.386 (1.241, 1.550)	<0.001	1.843 (1.661, 2.045)	<0.001	1.762 (1.555, 1.996)	<0.001
**Hyperlipidemia**								
No	1.000		1.000		1.000		1.000	
Yes	0.851 (0.777, 0.932)	0.001	0.917 (0.826, 1.017)	0.102	0.793 (0.721, 0.872)	<0.001	0.939 (0.830, 1.061)	0.312
**Hypertension**								
No	1.000		1.000		1.000		1.000	
Yes	0.786 (0.728, 0.848)	<0.001	0.955 (0.873, 1.044)	0.312	0.673 (0.621, 0.729)	<0.001	0.812 (0.731, 0.903)	<0.001
**Diabetes**								
No	1.000		1.000		1.000		1.000	
Yes	0.707 (0.635, 0.786)	<0.001	0.746 (0.662, 0.840)	<0.001	0.603 (0.538, 0.676)	<0.001	0.705 (0.611, 0.814)	<0.001

^a^Univariate linear regression model without adjustment.

^b^Multivariate logistic regression analyses adjustment for province, sex, age, BMI, education, smoking status, history of fracture, hyperlipidemia, hypertension, and diabetes as independent variables.

## Discussion

There are often no characteristic manifestations or only back pain in the early stage of the disease, and patients and even clinicians are prone to ignore the disease. Most patients are diagnosed only after passive physical examination or brittle fracture. Therefore, understanding the risk factors of osteoporosis is beneficial to the early diagnosis and prevention of osteoporosis.

China is the largest developing country in the world, constituting one-fifth of the global population and even higher percentage of the elderly population. The epidemiology of osteoporosis in China had its own unique features compared with that in Western countries. Nationwide studies on epidemiology of osteoporosis, especially in Chinese mainland are scarce. According to the results of 2020 population census of China released by the National Bureau of Statistics, people aged 60 or above accounts for 18.7% of the total population, which was only 13.26% in 2010, and the number of people aged 60 or above has been increased over 86 million in the past 10 years (14). Our study confirmed that aged 60 or above was an important risk factor of osteoporosis and osteopenia. According to the estimated prevalence of osteoporosis in our study, there would be more than 100 million aged people suffering osteoporosis, and the number will keep rising. However, a large number of patients with osteoporosis were still far from being fully evaluated and managed.

The respondents came from two municipalities, including Beijing and Shanghai, and five provinces across China including Guangzhou and Shenzhen in Guangdong province, Ganzhou in Jiangxi Province, Lanzhou in Gansu Province, Changchun in Jilin Province and Kunming in Yunnan Province. The distribution represents the north, east, south, west, north-east, south-east, and south-west regions of China with representative features in climate, environment and lifestyles. In recent overview of osteoporosis, a higher prevalence among residents in northern China was reported (15). And Zeng‘s study indicated that BMD values at the spine and hip were significantly lower in the participants from Southwest China than in those from other geographic regions (3). In our study, we included more regions of China, and found that the prevalence of osteoporosis of the inland regions including the southeast, the southwest, the northwest and the northeast were relatively higher than that of the eastern coastal regions. Multiple region-related factors, such as genetic predisposition, latitude and longitude (3), diet (16, 17), sunlight (18), physical activities (19), lifestyles (20), socioeconomic status (21), and even environment pollution (22, 23), could contribute to the differences in the prevalence of different regions of China.

Educational level is one of the most important socioeconomic indicators and closely related to the income level. People with higher education levels usually have stronger health awareness and more health knowledge, living in a healthier lifestyle with better medical resources. It contributes to the decreased prevalence of osteoporosis along with increased education level in the middle-aged and elderly people. Studies also had confirmed that knowledge awareness of osteoporosis was significantly correlated with BMD in postmenopausal women (5, 24).

Chronic metabolic diseases with high incidences in China including hypertension, hyperlipidemia and diabetes affect the development of osteoporosis physically (10–12). However, our study revealed a protective factor of diabetes on osteopenia and osteoporosis. Some large clinical studies (25, 26) also found that patients with diabetes had preserved or even increased BMD compared with control individuals without diabetes. The authors postulated that additional factors, including altered adipokine levels such as increased leptin and reduced adiponectin, and hyperinsulinemia might potentially have mediated the effects of obesity on BMD in diabetes. In addition, insulin resistance might also contribute to the reduced bone turnover and higher BMD in diabetes (27). These findings have been subsequently confirmed and extended by the meta-analysis to date on the association between BMD and diabetes. Despite higher levels of BMD than individuals who do not have diabetes, patients with diabetes tend to sustain fragility fractures with impaired bone material properties seeming to most consistently contribute to skeletal frailty in patients with diabetes. Alterations in bone material properties are at least partially related to glycation end products accumulation (28), which may be caused by the usage of insulin or other hypoglycemic drugs (29).

In addition to the influence of aging, region, education level, and history of chronic diseases, we also confirmed a higher prevalence in women and people with low BMI or a history of fracture which was consistent with previous findings (3, 13). The prevalence of osteoporosis in men over 60 years old is about one-third of that of women. The gradually decreased androgen levels associated with aging, decreased exposure to physical exercises and social activities may contribute to the occurrence of osteoporosis in the elderly men. In addition to decreased physical exercises and social activities, menopause occurs with the rapid decrease in estrogen level, which causes over activated osteoclast formation and imbalanced bone metabolism in the elderly women, and finally leads to dramatic bone loss (30). Also, a previous study suggested that nutrient deficiencies, such as vitamins, are common in older women (31). Low intake of daily protein and vitamins also contributes to bone dystrophy and accelerated bone loss.

According to the Chinese society of Osteoporosis and Mineral Research (CSOBMR) criteria, osteoporosis of postmenopausal women and man over 50 years old was defined as a *T*-score of BMD≤−2.5 SD (32). It was reported that the BMD values of lumbar spine were lower than that of hip in age and gender matched groups (3), and spine is the site of rapid bone loss compared with hip. The present study also confirmed that the prevalence of osteoporosis would be greatly reduced if diagnosing by BMD of each single region only. Also, the prevalence of osteoporosis was dramatically reduced according to BMD of hip. To screen out more patients with osteoporosis, it is more appropriate to make a diagnosis when *T*-score of BMD of any part≤ −2.5 SD.

In order to achieve the early diagnosis and prevention of osteoporosis, it is important to identify and pay close attention to the high-risk population of osteoporosis. In this study, the regional differences in osteoporosis prevalence in China were revealed. It was also proved that women, aged 60 or above, BMI lower than 18.5 kg/m^2^, low education level including middle school, primary school and no formal education as well as current regular smoking, a history of fracture are associated with a higher risk of osteoporosis and osteopenia in the middle-aged and elderly people of China. These findings are helpful to formulate the screening program and local policies for prevention and treatment of osteoporosis.

The present study had some strength. Firstly, compared to previous studies on osteoporosis prevalence in China (3, 33), we include some major demographic and socioeconomic factors, such as age, gender, education levels, history of chronic diseases, which were confirmed to be associated with the occurrence of bone loss and osteoporosis. So far, it was the first study to observe the relationship between these factors and the risk of osteoporosis at the same time in China. It provided basis of which more prevention and treatment resources should be invested into particular population exposed to specific factors. Secondly, the DXA instruments used in all centers were of the same model, and the BMD values of both lumbar spine and hip were collected. Therefore, the BMD reference database established in the present study was comparable, which was largely improved compared with the previous studies of BMD measurements in the Chinese population (3, 34, 35).

Nevertheless, there were also several limitations. First of all, the participants in our study were volunteers from multiple regions, but not randomly selected, which might cause selection bias. Secondly, the participants who were already on anti-osteoporosis treatment were not excluded, and it might cause a lower prevalence than it actually was. Third, considering the time costs and practical operability, some other factors, such as dietary intake, medical history and physical activities which might also affect BMD values at certain degree were not collected in the study.

## Conclusion

This multicenter survey revealed dramatic regional differences in osteoporosis prevalence in China, and woman, aged 60 years or older, low BMI, low education level, and current regular smoking might contribute to the occurrence of osteoporosis. More prevention and treatment resources should be invested into particular population exposed to these risk factors.

## Data availability statement

The original contributions presented in the study are included in the article/Supplementary material, further inquiries can be directed to the corresponding author.

## Ethics statement

The studies involving human participants were reviewed and approved by Institutional Review Board at Longhua Hospital affiliated to the Shanghai University of Traditional Chinese Medicine. The patients/participants provided their written informed consent to participate in this study.

## Author contributions

All authors contributed to the study's conception and design, as well as the acquisition, analysis, interpretation of data, drafting and revising of the article, and agreed to be accountable for all aspects of the work.

## The China Community-based Cohort of Osteoporosis (CCCO) collaborative group

(a) Central Steering Committee
  Yong-jun Wang, Bing Shu, De-zhi Tang, Chen-guang Li, Jing Wang.

(b) Study coordinating centers
*Shanghai*
  Longhua Hospital, Shanghai University of Traditional Chinese Medicine: Qi Shi, Xue-jun Cui, Qianqian-Liang, Yan Zhang, Yan-ping Yang, Sheng Lu.
*Gansu*
  Gansu Provincial Hospital of Traditional Chinese Medicine: Xing-wen Xie, Ding-peng Li, Jin Huang.
*Jiangxi*
  Ganzhou Nankang District Traditional Chinese Medicine Hospital: Zhang-yu Liao, Xian-hui Zeng, Wang Liao.
  The First People's hospital of Nankang District: Qing Zhang, Wei-jian Zhong, Zhuo-lin Hu.
*Yunnan*
  Yunnan Provincial Hospital of Traditional Chinese Medicine: Li-juan Jiang, Wen-jun Pu, Yuan Chai.
*Guangdong*
  The First Hospital Affiliated to Guangzhou University of Traditional Chinese Medicine: De Liang, Xiao-bing Jiang, Hui Ren, Wen-hua Zhao.
  Guangdong Provincial Hospital of Traditional Chinese Medicine: Bo-lai Chen, Yong-peng Lin, Yong-jin Li.
  Shenzhen Pingle Orthopedic Hospital: Bao-lin Li, Yong Zhang, Yin Lian.
*Beijing*
  Dongzhimen Hospital, Beijing University of Chinese Medicine: Xin-chao Lin, Qiao-hui Yang, Rong-lu Yang.
  Wangjing Hospital of China Academy of Chinese Medical Sciences: Xu Wei, Yan-ming Xie, Yi-li Zhang, Meng-hua Sun.
*Jilin*
  Hospital Affiliated to Changchun University of Traditional Chinese Medicine: Xiang-yang Leng, Zong-jian Luo, Hong-wei Gao.
