# Supplementary material for: The prevalence of osteoporosis in China, a community based cohort study of osteoporosis
Source: Front Public Health. 2023 Feb 16;11:1084005. doi: 10.3389/fpubh.2023.1084005 (PMC9978786; doi:10.3389/fpubh.2023.1084005)
Supplement: Supplementary file 1 [file Table_1.DOCX]

eTable1 Serum levels of bone metabolic markers and calcium and phosphorus metabolism indicators of the participants

|  | Mean (SD) | | | | | | | |
| --- | --- | --- | --- | --- | --- | --- | --- | --- |
|  | P, mmol/L  (n=19527) | Ca, mmol/L  (n=19531) | PINP, ng/ml  (n=19496) | β-CTX, ng/ml  (n=19482) | OST, ng/ml  (n=18574) | ALP, U/L  (n=18605) | PTH, pmol/L  (n=16105) | 25(OH)D, ng/ml  (n=19449) |
| Overall | 1.27 (0.54) | 2.33 (0.10) | 51.37 (22.23) | 0.36 (0.66) | 15.40 (6.68) | 79.55 (23.40) | 3.49 (1.77) | 20.67 (7.83) |
| Gender |  |  |  |  |  |  |  |  |
| Men | 1.15 (0.52) | 2.31 (0.10) | 41.71 (17.71) | 0.34 (0.78) | 12.58 (5.19) | 75.38 (21.73) | 3.41 (1.62) | 21.28 (7.76) |
| Women | 1.32 (0.54) | 2.34 (0.10) | 55.23 (22.68) | 0.37 (0.61) | 16.51 (6.87) | 81.20 (23.82) | 3.52 (1.82) | 20.42 (7.84) |
| *P* value | <0.001 | <0.001 | <0.001 | <0.001 | <0.001 | <0.001 | <0.001 | <0.001 |
| Age |  |  |  |  |  |  |  |  |
| 40-49 years | 1.28 (0.41) | 2.35 (0.10) | 53.71 (22.22) | 0.30 (0.16) | 14.27 (5.38) | 76.13 (24.93) | 3.13 (1.27) | 19.52 (7.09) |
| 50-59 years | 1.33 (0.56) | 2.35 (0.10) | 55.94 (22.85) | 0.34 (0.44) | 16.26 (7.19) | 79.46 (22.24) | 3.32 (2.01) | 21.12 (7.79) |
| 60-69 years | 1.26 (0.52) | 2.33 (0.10) | 50.20 (21.47) | 0.36 (0.69) | 15.28 (6.44) | 79.54 (23.26) | 3.52 (1.63) | 20.62 (7.73) |
| 70-79 years | 1.22 (0.56) | 2.32 (0.10) | 48.33 (22.4) | 0.39 (0.81) | 14.62 (6.46) | 80.20 (25.2) | 3.69 (1.74) | 20.35 (8.08) |
| 80- years | 1.22 (0.44) | 2.32 (0.09) | 44.65 (17.93) | 0.42 (1.13) | 13.93 (5.88) | 72.74 (20.68) | 3.48 (1.65) | 18.79 (8.66) |
| *P* value | <0.001 | <0.001 | <0.001 | <0.001 | <0.001 | <0.001 | <0.001 | <0.001 |
| BMI, kg/m^2^ |  |  |  |  |  |  |  |  |
| <18.5 | 1.32 (0.52) | 2.33 (0.10) | 57.05 (30.70) | 0.39 (0.62) | 17.96 (7.92) | 78.78 (33.95) | 3.36 (1.51) | 21.96 (8.91) |
| 18.5-24.9 | 1.28 (0.53) | 2.33 (0.10) | 52.15 (22.82) | 0.38 (0.72) | 16.03 (7.03) | 79.11 (22.87) | 3.45 (1.82) | 21.07 (7.99) |
| ≥ 25.0 | 1.27 (0.56) | 2.33 (0.10) | 49.53 (20.15) | 0.33 (0.59) | 14.09 (5.67) | 80.21 (23.15) | 3.55 (1.70) | 19.75 (7.33) |
| *P* value | <0.001 | 0.574 | <0.001 | <0.001 | <0.001 | <0.001 | <0.001 | <0.001 |
| Education |  |  |  |  |  |  |  |  |
| No formal education | 1.28 (0.61) | 2.33 (0.09) | 58.13 (24.95) | 0.38 (0.33) | 17.23 (9.83) | 86.99 (23.78) | 3.72 (1.86) | 21.64 (8.08) |
| Primary school | 1.29 (0.66) | 2.33 (0.09) | 53.46 (23.94) | 0.34 (0.39) | 15.57 (6.44) | 82.48 (24.4) | 3.52 (1.59) | 21.48 (7.84) |
| Middle school | 1.27 (0.52) | 2.33 (0.10) | 50.33 (21.56) | 0.37 (0.75) | 15.15 (6.24) | 80.25 (23.85) | 3.55 (1.69) | 19.99 (7.68) |
| High school | 1.30 (0.53) | 2.33 (0.10) | 51.73 (21.43) | 0.35 (0.67) | 15.64 (6.84) | 78.31 (22.94) | 3.38 (1.93) | 20.43 (7.87) |
| College | 1.25 (0.49) | 2.33 (0.10) | 49.07 (22.60) | 0.36 (0.72) | 14.81 (5.78) | 76.17 (21.99) | 3.42 (1.63) | 20.55 (7.73) |
| University or higher | 1.21 (0.45) | 2.33 (0.09) | 46.93 (20.01) | 0.42 (0.92) | 14.15 (6.88) | 75.13 (21.32) | 3.64 (1.90) | 20.34 (7.28) |
| *P* value | <0.001 | 0.012 | <0.001 | <0.001 | <0.001 | <0.001 | <0.001 | <0.001 |
| Region |  |  |  |  |  |  |  |  |
| Shanghai | 1.10 (0.19) | 2.29 (0.10) | 47.40 (19.87) | 0.53 (1.19) | 16.36 (6.42) | 77.47 (20.45) | 4.20 (1.73) | 21.25 (7.58) |
| Beijing | 1.39 (0.33) | 2.35 (0.09) | 49.24 (21.30) | 0.27 (0.12) | 13.45 (6.03) | 79.01 (21.94) | 3.20 (1.50) | 16.92 (6.58) |
| Guangdong | 1.45 (0.50) | 2.34 (0.10) | 53.68 (24.06) | 0.29 (0.18) | 15.68 (6.35) | 70.29 (20.95) | 2.70 (1.25) | 26.43 (6.46) |
| Jilin | 1.27 (0.40) | 2.35 (0.08) | 51.61 (20.87) | 0.25 (0.12) | 14.47 (5.32) | 81.73 (24.33) | 2.89 (1.25) | 17.15 (6.45) |
| Gansu | 1.17 (0.68) | 2.34 (0.10) | 53.54 (23.12) | 0.26 (0.12) | 15.84 (7.60) | 89.27 (25.67) | 3.94 (2.15) | 15.40 (6.43) |
| Yunnan | 1.57 (1.10) | 2.36 (0.09) | 53.19 (22.18) | 0.36 (0.17) | 14.45 (6.85) | 81.80 (24.47) | 4.56 (2.26) | 21.12 (6.52) |
| Jiangxi | 1.2 (0.32) | 2.35 (0.09) | 58.24 (24.50) | 0.34 (0.18) | 16.53 (7.51) | 84.83 (24.27) | 3.11 (1.66) | 25.08 (6.87) |
| P value | <0.001 | <0.001 | <0.001 | <0.001 | <0.001 | <0.001 | <0.001 | <0.001 |
| Hyperlipidemia |  |  |  |  |  |  |  |  |
| Yes | 1.27(0.56) | 2.33(0.10) | 52.32(22.92) | 0.37(0.65) | 15.81(7.01) | 79.89(23.62) | 3.56(1.85) | 21.13(7.82) |
| No | 1.3(0.52) | 2.34(0.10) | 47.96(19.64) | 0.37(0.8) | 14.36(5.64) | 78.22(23.16) | 3.39(1.58) | 20.29(7.82) |
| P value | <0.001 | <0.001 | <0.001 | <0.001 | <0.001 | <0.001 | <0.001 | <0.001 |
| Hypertension |  |  |  |  |  |  |  |  |
| Yes | 1.28(0.54) | 2.33(0.10) | 52.55(22.81) | 0.36(0.65) | 15.8(6.74) | 78.71(23.01) | 3.44(1.76) | 21.26(7.79) |
| No | 1.26(0.56) | 2.33(0.10) | 49.45(21.50) | 0.38(0.74) | 14.92(6.78) | 81.1(24.30) | 3.69(1.85) | 20.31(7.85) |
| P value | <0.001 | 0.703 | <0.001 | 0.842 | <0.001 | <0.001 | <0.001 | <0.001 |
| Diabetes |  |  |  |  |  |  |  |  |
| Yes | 1.28(0.56) | 2.33(0.10) | 52.53(22.38) | 0.36(0.65) | 15.76(6.73) | 79.35(23.18) | 3.52(1.79) | 20.61(7.82) |
| No | 1.24(0.47) | 2.34(0.10) | 42.62(18.83) | 0.36(0.81) | 12.42(5.64) | 79.74(24.90) | 3.27(1.64) | 19.87(7.51) |
| P value | 0.027 | <0.001 | <0.001 | <0.001 | <0.001 | 0.510 | <0.001 | <0.001 |
| Bone mass category |  |  |  |  |  |  |  |  |
| Osteoporosis | 1.31 (0.59) | 2.34 (0.10) | 57.74 (24.03) | 0.37 (0.49) | 17.13 (7.67) | 84.17 (25.93) | 3.54 (1.84) | 20.61 (8.04) |
| Osteopenia | 1.27 (0.53) | 2.33 (0.10) | 50.22 (20.71) | 0.35 (0.65) | 15.03 (5.74) | 78.51 (21.86) | 3.45 (1.60) | 20.56 (7.80) |
| Normal | 1.21 (0.44) | 2.32 (0.10) | 43.51 (18.92) | 0.36 (0.88) | 13.35 (5.87) | 74.15 (20.33) | 3.49 (1.94) | 20.96 (7.52) |
| P value | <0.001 | <0.001 | <0.001 | <0.001 | <0.001 | <0.001 | 0.055 | 0.002 |

eTable2. Estimated prevalence of osteoporosis and osteopenia in each group by sexual

|  | Osteopenia  (n=8330) | | Osteoporosis  (n=7051) | |
| --- | --- | --- | --- | --- |
|  | Men (n=2464) | Women (n=5866) | Men (n=1151) | Women (n=5900) |
| Age |  |  |  |  |
| 40-49 years | / | 42.94(36.17,49.71) | / | 19.26(13.85,24.67) |
| 50-59 years | 48.4(45.34,51.45) | 45.95(44.48,47.42) | 23.47(20.88,26.06) | 30.07(28.71,31.42) |
| 60-69 years | 43.71(41.88,45.53) | 41.22(40.03,42.41) | 19.1(17.64,20.55) | 43.46(42.27,44.65) |
| 70-79 years | 39.96(37.5,42.42) | 35.16(33.34,36.99) | 20.99(18.95,23.04) | 54.66(52.76,56.56) |
| 80- years | 38.98(26.16,51.8) | 32.53(22.24,42.82) | 23.73(12.55,34.91) | 60.24(49.49,70.99) |
| BMI |  |  |  |  |
| <18.5kg/m^2^ | 32.09(22.76,41.42) | 25.26(20.88,29.64) | 54.13(44.34,63.92) | 70.3(65.68,74.91) |
| 18.5-24.9 kg/m^2^ | 47.11(45.18,49.04) | 42.76(41.63,43.89) | 25.77(24.06,27.48) | 42.66(41.54,43.77) |
| ≥ 25.0 kg/m^2^ | 42.55(40.41,44.69) | 44.8(43.36,46.24) | 12.43(10.99,13.87) | 26.49(25.25,27.73) |
| Education |  |  |  |  |
| No formal education | 38.86(28.4,49.31) | 34.18(30.87,37.49) | 17.95(9.37,26.54) | 55.73(52.28,59.18) |
| Primary school | 45.4(41.58,49.22) | 38.37(36.16,40.57) | 22.41(19.18,25.63) | 48.66(46.4,50.92) |
| Middle school | 47.32(44.78,49.87) | 44.14(42.52,45.76) | 19.01(17.01,21.01) | 36.28(34.73,37.82) |
| High school | 45.83(43.09,48.58) | 44.47(42.98,45.96) | 19.69(17.49,21.9) | 33.47(32.07,34.87) |
| College | 42.22(38.28,46.16) | 45.03(42.06,47.99) | 19.18(16.07,22.3) | 32.7(29.98,35.42) |
| University or higher | 40.47(36.1,44.84) | 44.76(40.43,49.09) | 21.84(18.07,25.61) | 36.74(32.6,40.87) |
| Region |  |  |  |  |
| Shanghai | 37.67(35.23,40.11) | 43.7(42.07,45.33) | 11.70(10.06,13.33) | 31.52(30.03,33) |
| Beijing | 47.89(44.07,51.71) | 45.14(42.83,47.45) | 16.09(13.32,18.86) | 33.71(31.57,35.86) |
| Guangdong | 48.05(44.43,51.67) | 44.59(42.58,46.59) | 20.41(17.51,23.32) | 34.7(32.81,36.59) |
| Jilin | 45.89(40.86,50.92) | 42.44(39.45,45.43) | 19.01(15.17,22.86) | 38.55(35.67,41.43) |
| Gansu | 47.92(43.98,51.85) | 46.74(44.43,49.04) | 23.16(19.77,26.55) | 35.6(33.43,37.77) |
| Yunnan | 48.71(44.61,52.82) | 35.79(32.69,38.89) | 29.76(26.04,33.49) | 54.94(51.74,58.13) |
| Jiangxi | 42.84(38.06,47.61) | 31.35(28.54,34.16) | 38.67(34.02,43.32) | 60.42(57.48,63.37) |
| Smoking status |  |  |  |  |
| Never | 42.7(40.63,44.78) | 42.79(41.84,43.74) | 21.56(19.8,23.32) | 38.78(37.86,39.70) |
| Former | 45.93(42.45,49.41) | 37.85(27.54,48.16) | 16.56(14.05,19.06) | 38.91(28.41,49.40) |
| Current regular | 47.46(44.96,49.97) | 44.51(38.07,50.95) | 23.01(20.9,25.12) | 37.63(31.50,43.76) |
| Passive smoking | 47.5(39.27,55.74) | 43.41(40.32,46.50) | 16.04(9.84,22.24) | 37.95(34.97,40.93) |
| History of fracture | 49.34(45.91,52.78) | 40.74(38.75,42.73) | 21.96(19.09,24.83) | 45.87(43.87,47.87) |
| Hyperlipidemia |  |  |  |  |
| Yes | 43.50(40.12,46.88) | 42.27(40.34,44.20) | 16.35(13.80,18.90) | 35.31(33.47,37.14) |
| No | 44.95(43.41,46.49) | 42.82(41.85,43.79) | 21.62(20.34,22.91) | 38.69(37.75,39.63) |
| Hypertension |  |  |  |  |
| Yes | 44.13(41.80,46.46) | 42.81(41.30,44.33) | 14.18(12.52,15.85) | 35.65(34.21,37.09) |
| No | 44.99(43.24,46.74) | 42.68(41.63,43.73) | 24.00(22.50,25.50) | 39.05(38.03,40.07) |
| Diabetes |  |  |  |  |
| Yes | 40.70(36.99,44.42) | 41.58(38.96,44.20) | 14.18(11.52,16.85) | 33.97(31.49,36.45) |
| No | 45.28(43.77,46.79) | 42.84(41.92,43.76) | 21.67(20.41,22.92) | 38.49(37.60,39.37) |
